# Intra-Arterial Infusion Chemotherapy in Advanced Pancreatic Cancer: A Comprehensive Review

**DOI:** 10.3390/cancers14020450

**Published:** 2022-01-17

**Authors:** Carmelo Laface, Mariarita Laforgia, Pasquale Molinari, Caterina Foti, Francesca Ambrogio, Cosmo Damiano Gadaleta, Girolamo Ranieri

**Affiliations:** 1 Interventional and Medical Oncology Unit, IRCCS Istituto Tumori “Giovanni Paolo II”, Viale Orazio Flacco 65, 70124 Bari, Italy; c.laface@studenti.uniba.it (C.L.); p.molinari@oncologico.bari.it (P.M.); c.gadaleta@oncologico.bari.it (C.D.G.); 2 Department of Biomedical Sciences and Clinical Oncology, University of Bari Aldo Moro, 70124 Bari, Italy; 3 Pharmacy Unit, IRCCS Istituto Tumori “Giovanni Paolo II”, Viale Orazio Flacco 65, 70124 Bari, Italy; m.laforgia@oncologico.bari.it; 4 Department of Biomedical Science and Human Oncology, Dermatological Clinic, University of Bari Aldo Moro, 70124 Bari, Italy; caterina.foti@uniba.it (C.F.); francesca.ambrogio@uniba.it (F.A.)

**Keywords:** pancreatic cancer, intra-arterial infusion chemotherapy, implanted pump or port

## Abstract

**Simple Summary:**

Pancreatic cancer has a very poor prognosis. The few available therapeutic options are characterized by low efficacy and high toxicity due to the intrinsic chemoresistance of this tumor type. To improve clinical results, some clinical trials have evaluated regional chemotherapy as a treatment option for PC. The pancreatic arterial infusion of chemotherapeutics has the aim of obtaining higher local concentrations of drugs and, at the same time, of limiting systemic toxicity. This therapeutic approach has already been successfully evaluated for the treatment of several types of tumors. Regarding advanced pancreatic cancers, only a few clinical studies have investigated the safety and efficacy of this treatment, with very promising results. Therefore, in this review, we summarize literature data on the clinical approaches to pancreatic arterial drug administration for the treatment of advanced PC to deepen knowledge on this topic.

**Abstract:**

Advanced pancreatic cancer (PC) has a very poor prognosis due to its chemoresistant nature. Nowadays, only a few therapeutic options are available for PC, and the most effective ones are characterized by low response rates (RRs), short progression-free survival and overall survival, and severe toxicity. To improve clinical results, small series studies have evaluated loco-regional chemotherapy as a treatment option for PC, demonstrating its dose-dependent sensitivity towards the tumor. In fact, pancreatic arterial infusion (PAI) chemotherapy allows higher local concentrations of chemotherapeutic agents, sparing healthy tissues with a lower rate of adverse events compared to systemic chemotherapy. This therapeutic approach has already been evaluated in different types of tumors, especially in primary and metastatic liver cancers, with favourable results. With regard to advanced PC, a few clinical studies have investigated the safety and efficacy of PAI with promising results, especially in terms of RRs compared to systemic chemotherapy. However, clear evidence about its efficacy has not been established yet nor have the underlying mechanisms leading to its success. In this review, we aim to summarize the literature data on the clinical approaches to pancreatic arterial drug administration in terms of techniques, drug pharmacokinetics, and clinical outcomes for advanced PC.

## 1. Introduction

Pancreatic cancer (PC) is the fourth most common cause of cancer mortality, with a 5-year survival rate of only 8%, although it is considered a rare tumor [1,2,3]. Standard therapies are represented by radical surgery and adjuvant chemotherapy for the early stages, although 60–70% of patients present a relapse after 2 years [4]. Concerning metastatic setting, a randomized phase II-III French study published in 2011 evaluated 342 previously untreated advanced PC patients (age 18–75 years, ECOG 0-1) that were randomized to receive the FOLFIRINOX schedule (5-fluorouracil, folic acid, irinotecan, oxaliplatin) vs. gemcitabine [5]. The results showed f a significant advantage or the experimental group both in the median progression-free survival (mPFS) (6.4 months vs. 3.3 months; HR 0.47 (0.37–0.59); *p* < 0.0001) and in the median overall survival (mOS) (11.1 months vs. 6.8 months; HR 0.57 (0.45–0.73); *p* = 0.001). However, several Grade 3–4 adverse events (AEs) occurred in the experimental group patients: 23% experienced asthenia, 15% experienced vomiting, 13 % experienced diarrhoea, and 9% experienced peripheral neuropathy [5]. More recently, a phase III study evaluated 154 advanced PC patients with BRCA 1–2 germline mutation and without disease progression during the 4 months after first-line platinum-based chemotherapy to receive olaparib or placebo [6]. This clinical trial demonstrated that the mPFS was significantly longer in the olaparib group than it was in the control group (median 7.4 months versus 3.8 months, respectively) [6]. Gemcitabine plus nab-paclitaxel is another schedule for the treatment of fit and untreated advanced PC patients; a multicentre phase III study showed that this combination regimen is associated with a longer mPFS (median 5.5 months vs. 3.7 months; HR 0.69 (0.62–0.83); *p* < 0.0001) and OS (median 8.5 months vs. 6.7 months; HR 0.72 (0.62–0.83); *p* < 0.0001) compared to gemcitabine alone [7]. As a further development in these treatments, another recent schedule for fit patients is the quadruple combination therapy, called PAXG (cisplatin 30 mg/m^2^, nab-paclitaxel 150 mg/m^2^, and gemcitabine 800 mg/m^2^ on days 1 and 15 and oral capecitabine 1250 mg/m^2^ on days 1–28 every 4 weeks), including all drugs indicated in PC. PACT-19 is a randomised phase 2 trial comparing PAXG to the standard combination of nab-paclitaxel and gemcitabine. At 6 months, 74% of the participants in the experimental group were alive and free from disease progression compared to 46% of the patients in the control group [8].

PC generally has a very poor prognosis because only a few therapeutic options are available; moreover, it has low efficacy and high toxicity [9,10,11].

The former is due to the drug resistance of PC, which mainly depends on the simultaneous presence of a mechanical and a biological barrier [12]. The first is represented by a very dense, poorly vascularized, fibrotic, almost drug-impenetrable envelope surrounding pancreatic tumor area [13,14,15,16,17] even though the pancreas itself has a poor vascularization. This condition hampers the systemic chemotherapeutic agents to reach the pancreas and, therefore, the tumor cells in a sufficient amount to be effective [13]. The second impediment derives from the high expression of the cells comprising the multidrug resistance gene (MDR1) product, the membrane-bound P-170 glycoprotein [18]. It is a part of an ATP-dependent drug efflux enzyme system that is able to quickly eliminate chemotherapeutic drugs from tumor cells [18]. In this regard, non-randomized, small series studies have demonstrated that PC has a dose-dependent sensitivity to regional chemotherapy [19,20,21,22]. In fact, it is expected that the drug dose that is delivered to the pancreatic tumor site must be at least five-fold higher to overcome the tumor cell resistance due to the P-170 glycoprotein [23].

In summary, the poor prognosis, the limited treatment strategies, and the anatomical and biological features of PC justify the medical need for new therapeutic options. One recent promising route is the application of loco-regional therapies, particularly the pancreatic arterial infusion (PAI) of chemotherapy, delivering remarkably higher concentrations of antineoplastic agents to the tumor site than systemic administration and limiting adverse events (AEs).This type of therapeutic approach has already been successfully evaluated in various solid tumors, specifically in primary and metastatic liver cancers [24,25,26,27,28,29,30,31,32,33,34,35,36]. As for advanced PC, only a few clinical trials have investigated the safety and efficacy of PAI with promising results, especially in terms of high response rates (RRs) compared to systemic chemotherapy [23]. However, clear evidence about its efficacy has not been established yet nor have the biological mechanisms underlying clinical response.

In this review, we aim to summarize the literature data on the therapeutic approaches to the pancreatic arterial administration of chemotherapy in terms of techniques, drug pharmacokinetics, clinical studies, and clinical results for advanced PC.

## 2. Pancreatic Arterial Infusion of Chemotherapy

### 2.1. Technical Procedure

**Figure 1:**
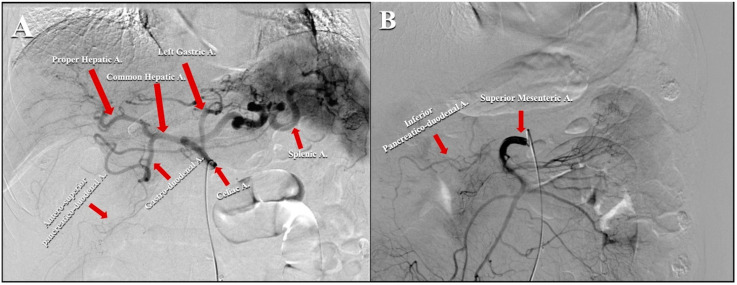
(**A**) Angiographic scan representing the vascular anatomy of celiac artery. (**B**) Angiographic scan representing vascular anatomy of superior mesenteric artery.

The pancreas is characterized by an extremely variable vascular anatomy and is supplied by several vessels [37]. Specifically, pancreatic parenchyma is vascularized by two main vessels: the celiac artery and the superior mesenteric artery (SMA) [38]. The first feeds the pancreatica magna, dorsal pancreatic, and caudal pancreatic arteries, supplying the pancreatic body and tail [38,39,40]. On the other hand, the pancreatic head is supplied by the pancreaticoduodenal arcade that derives from the junction of the anterosuperior pancreaticoduodenal and the postero-superior-pancreatico-duodenal arteries that originate from the gastroduodenal and the inferior pancreaticoduodenal ones that arise from the SMA [38] (Figure 1A,B).

This peculiar anatomy makes the catheter placement site one of the main issues in PAI chemotherapy. The first studies reported PAI through both the celiac artery and SMA to cover all pancreatic cancer areas [41]. For this reason, PAI chemotherapy is considered to be a difficult challenge due to the complicated management of dual arterial infusion. Moreover, a pilot clinical study of arterial drug infusion via the SMA in the context of dual PAI for advanced PC reported gastrointestinal toxicity, such as hypoalbuminemia and diarrhoea [42].

**Figure 2:**
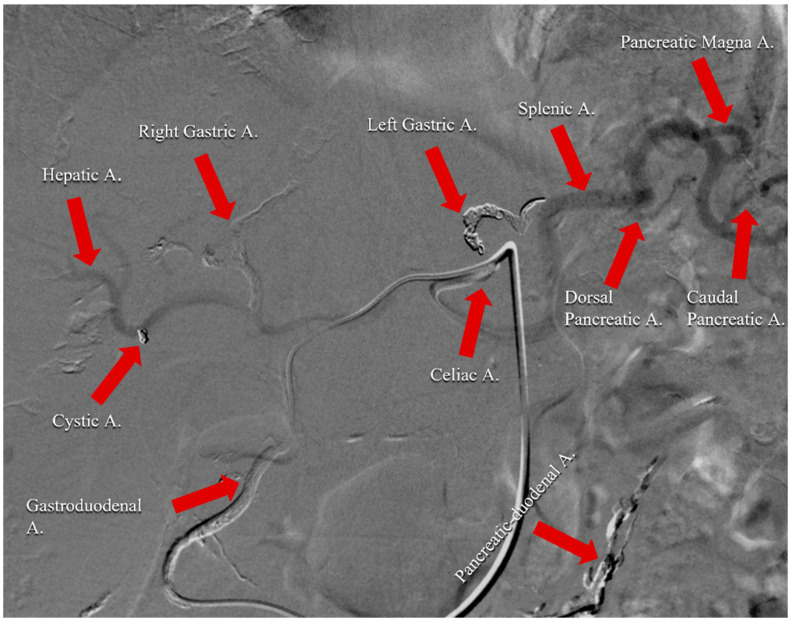
Angiographic scan following injection of contrast medium through celiac arterial after the completion of the technical procedure. It represents an example of the vascular remodulation of the celiac trunk for a patient affected by pancreatic cancer with liver metastases. Right gastric, left gastric, gastroduodenal, cystic, and pancreatic-duodenal arteries were embolized with spirals and coils to avoid perfusion of the stomach, duodenum, and gallbladder. Infusion catheter was fixed into gastroduodenal artery.

Subsequent studies have described a new technique that consists of the unification of the two pancreatic vascular networks [43,44]. In this way, a single arterial infusion of chemotherapeutic drugs via the celiac artery has been achieved with a sufficient antitumor effect and without the AEs deriving from chemotherapeutic administration via the SMA. To be specific, this approach involves the embolization of all pancreatic arteries originating from the SMA to grant a single supply from the celiac artery. Concerning the arterial infusion technique, the first step consists of an angiographic examination using a 5.5-French angiographic catheter through both the celiac artery and SMA to evaluate pancreatic vascular anatomy. In the next step, a microcatheter is inserted into the pancreatic arteries arising from SMA: the anteroinferior pancreaticoduodenal and the posteroinferior pancreaticoduodenal arteries and their subsequent embolization employing microcoils. Sometimes, the dorsal pancreatic artery born from the SMA or inferior pancreaticoduodenal and the middle colic ones are also embolized. The embolization of the left and right gastric arteries and the right gastroepiploic artery is also performed to avoid the perfusion of the stomach and to increase the drug supply to the tumor. At this point, a 5-French catheter with a side hole is inserted through the femoral artery. For tumors of the pancreatic body or tail, the catheter tip is positioned into the hepatic artery with the side hole placed in the celiac artery to increase blood flow to the splenic artery. In the cases of tumors of the pancreatic head, the catheter tip is placed into the splenic artery, with the side hole in the celiac artery to increase flow to the gastroduodenal artery. Subsequently, the splenic artery is embolized with coils, and the infusion catheter is fixed. If the dorsal pancreatic artery arises from the splenic artery, the coils are inserted at the distal tract of dorsal pancreatic artery, allowing PAI into both the dorsal pancreatic and the gastroduodenal arteries. Moreover, in this way, the whole liver is successfully supplied by the celiac artery; this is a very useful strategy because the liver is the most frequent site for distant metastases in PC patients. Finally, the catheter’s proximal end is connected to a subcutaneous implantable port. CT angiographic scans are performed using the arterial infusion of the contrast medium through the implanted port. The procedure is considered successful when the whole tumor area is enhanced after the injection of the contrast medium through the celiac arterial and after the blood supply from the SMA disappears [41] (Figure 2).

However, in spite of these great advances in techniques, PC often invades the surrounding organs, and new networks of blood vessels are formed accordingly. Therefore, the unification of the pancreatic blood supply might also be complex in some cases because of the site and area of tumor invasion [43].

### 2.2. Pharmacokinetic Evaluation

The current knowledge about the pharmacokinetic data for PAI infusion is very poor. Only a few studies have tried to evaluate the advantage of PAI chemotherapy in terms of kinetics, but most of them concern animal model experiments.

Gemcitabine is one of the most used drugs in the treatment of advanced PC [45]. From a pharmacological point of view, it is a pyrimidine analogue that needs to be activated in the cells by the deoxycytidine kinase (dCK) enzyme in difluorodeoxycitidin-diphosphate and triphosphate [46]. Gemcitabine acts by altering the correct DNA synthesis through the inhibition of the ribonucleotide reductase enzyme [45]. Literature data have demonstrated that high levels of dCK are present in different human tumoral cells; therefore, PAI chemotherapy might significantly spare healthy tissues. The half-life of gemcitabine is 42–92 min after intravenous administration, but its pharmacokinetic properties have also been tested after hepatic arterial infusion (HAI), with interesting results in terms of liver extraction rates [47]. A study has investigated the feasibility of PAI with gemcitabine for the treatment of 10 Beagle dogs affected by locally advanced PC. The authors evaluated the drug concentration in blood and various tissue samples after the administration of the same dosage of gemcitabine (45 mg/kg) both through PAI via the celiac axis and SMA and intravenous infusion. The results demonstrated that PAI with gemcitabine is feasible and might significantly increase the drug concentration in serum and in the pancreatic tumor area, prolonging drug retention in the animal body with respect to intravenous infusion [48].

The compound 5-fluorouracil (5-FU) blocks both the conversion of cytosine nucleosides into the deoxy-derivatives and the incorporation of the thymidine nucleotide into the DNA strand [49]. The half-life of 5-FU is 8–20 min; therefore, its principal administration route is via continuous infusion (c.i.). Moreover, the infusion of an initial bolus allows the therapeutic window to be reached more rapidly, which is then sustained by the subsequent c.i. Literature data have already evaluated the 5-FU liver extraction rate after HAI, which is approximately 75–80% [24], and several clinical trials have demonstrated remarkable efficacy in the treatment of primary and secondary hepatic malignancies. Tao et al. tested the pharmacokinetic differences between PAI and the intravenous infusion of 5-FU in a Wistar rat model. In this study, the authors demonstrated that the maximum concentration of 5-FU in the pancreas as well as the clearance time of the pancreas were significantly higher in the population treated with PAI 5-FU (20.00 mg/g and 90 min for PAI versus 8.42 mg/g and 50 min for intravenous infusion) [50]. Other experimental studies employing dog and pig models of advanced PC confirmed that PAI 5-FU allows a higher drug concentration in PC without inducing toxicity on the healthy pancreatic tissues, the duodenum, and the liver compared to intravenous administration [44,51].

Platinum salts are alkylating agents that act on the N7 guanines along the DNA double strand. Intra-arterial hepatic versus the intravenous administration of cisplatin or oxaliplatin in a VX2 tumor model in White New Zealand female rabbits was performed (cisplatin 4 mg/kg or oxaliplatin 6 mg/kg). Atomic absorption spectrometry measured the platinum concentration at different times. The results demonstrated that oxaliplatin has better pharmacokinetic parameters and a more major tissue concentration via HAI than ev administration; for cisplatin, no differences in the pharmacokinetic parameters or platinum tissue accumulation were reported. No PAI kinetic studies in animals have been found in literature for platinum salts [52].

Only one study evaluated the pharmacokinetic parameters of PAI chemotherapy in a human population. To be specific, Kakizaki et al. enrolled four patients with advanced PC who underwent PAI Cisplatin with angiotensin II through an implantable drug delivery system. The authors showed that the drug concentration was 1.3 times higher in the PC tissue than it was in the adjacent normal tissue [53].

## 3. Clinical Trials

### 3.1. Published Reports

In the literature, only a few clinical trials have evaluated the role of PAI chemotherapy for advanced PC patients (UICC stage III or IV for liver metastases). This review investigates the safety concerning AEs and the efficacy in terms of the disease control rate (DCR), objective response rate (ORR), mPFS, and mOS of this type of loco-regional therapy. We have reported and summarized (Table 1) all of the clinical trials that have been performed.

#### 3.1.1. PAI as First Line Treatment without Systemic Chemotherapy

In 2000, Cantore et al. [54] showed the clinical outcomes for 96 never treated PC patients (48 with UICC stage III and 50 with UICC stage IV) who underwent a PAI with FLEC regimen every 3 weeks for 3 cycles (5-FU 1000 mg/m^2^, folinic acid 100 mg/m^2^, epirubicin 60 mg/m^2^, carboplatin 300 mg/m^2^) in a phase II clinical trial. From a pharmaceutical point of view, the choice of carboplatin with respect to cisplatin was strategically linked to its major solubility in an aqueous solution, translating into a higher drug dosage with a smaller administration volume. Each cycle was performed using an angiographic catheter that was placed into the gastroduodenal artery for pancreatic head tumors, into the splenic artery for pancreatic body and tail ones, and into the hepatic artery when liver metastases were present (50/96 patients), allowing half of the total dose was infused to the liver. When this technical approach was not possible, the chemotherapeutics were administered into the celiac axis. The authors reported an ORR of 15%, a DCR of 59%, and a mOS of 9.9 months (10.6 and 6.8 for stage III and IV, respectively). As far as toxicity was concerned, 25% of the patients experienced grade 3–4 hematologic and 3% experienced gastrointestinal AEs. Only one patient experienced a technique-related complication that consisted of an intimal dissection of the iliac artery.

In 2000, Homma et al. [55] analyzed PAI chemotherapy in 23 metastatic PC patients by means of an infusion catheter placed into the splenic artery for the treatment of primary PC as well as into the common hepatic artery for those patients with liver metastases in a phase II study. In the first cases, 5-FU (250 mg/m^2^ in 24 h c.i. for 7 days) and cisplatin (10 mg/m^2^ on Days 1, 3 and 5) administration was performed at weeks 1 and 3, within a course of 28 days. In the second cases, the same dosage of 5-FU was administered to both the primary tumor and metastatic lesions. The ORR was 73.9%, while the mean OS was 19 months. The ORR was 68.8% in the group of patients with liver metastases (16 patients), while the mean OS was 16.25 ± 8.35 months. About 20% of the patients experienced technique-related complications, such as dislocation of the catheter tip, arterial obstruction, and abscess in the femoral region, while no grade 3–4 AEs occurred.

In 2004, Cantore et al. [56] compared standard intravenous gemcitabine (32 with stage III and 35 with stage IV patients) with a PAI FLEC schedule (35 with stage III and 36 with stage IV patients) using an angiographic catheter placed into the celiac axis in a multicenter, open, randomized phase III clinical trial. Patients in the control group were administered 1000 mg/m^2^ in 30 min intravenous infusion every week a total of seven consecutive times followed by 1 week of rest and then received intravenous infusion weekly for 3 weeks every month. The ORR was 14% versus 5.9% in the FLEC and gemcitabine groups (no statistical difference), respectively, while the mOS was significantly longer in the experimental group (7.9 months versus 5.8 months (*p* = 0.036)). A total of 22.4% of patients in the gemcitabine group and 47.9% of the patients in the experimental group experienced at least one grade 3–4 AE.

In 2005, Aigner et al. [57] evaluated PAI via the celiac axis or common hepatic artery with fixed dose boli of mitomycin (10–15 mg), mitoxantrone (10 mg), and cisplatin (50 mg) adsorbed on degradable starch microspheres (3 mL; Spherex) for five cycles followed by one course of isolated hypoxic abdominal perfusion with mitomycin (30 mg) and cisplatin (70 mg) to prevent or treat potential peritoneal carcinosis in a phase II study involving 265 patients (112 with UICC stage III and 153 with IV). The mOS was 9 months, and the resecability rate for long-term survivors (>12 months) after treatment was 39%. No severe toxicity was reported.

In 2006, Mambrini et al. [58] tested a PAI FLEC regimen once more via the celiac axis in 211 patients (99 patients with UICC stage III and 112 with IV). The ORR, DCR, and mOS were 7.6%, 58.3%, and 9.2 months, respectively. No angiographic procedure-related complications were observed, although three intimal dissections of the iliac artery were reported. Among patients, 24% and 3% experienced Grade 3–4 hematological and gastrointestinal AEs, respectively; moreover, grade 3 alopecia was reported in 15% of the participants.

In 2007, Ishikawa et al. [59] conducted a clinical trial in 20 metastatic PC patients, testing PAI of gemcitabine, 5-FU, and cisplatin mixed together with angiotensin-II (AT-II). The aim was to increase the blood flow towards tumor areas while sparing healthy tissue. AT-II is one of the most powerful vasoconstrictors and is able to restrict blood flow in favor of liver perfusion during short (3–4 min) intra-arterial administrations. The ORR was 50%, while the mOS was 1 year. No severe AEs were reported.

In 2007, Tanaka et al. [42] tested the pancreatic arterial c.i. of 5-FU (333 mg/m^2^ on days 1–5 a week for 5 weeks) in association with radiotherapy (50 Gy at 2.0 Gy per fraction) in 20 patients (10 with UICC stage III and 10 with IV) in a pilot study. PAI was performed using one or two catheters placed into the pancreatic arteries according to angiographical evaluation. The ORR and mOS were 70% s and 11 months, respectively. Among the patients, 55% experienced severe non-hematological AEs, such as nausea and vomiting, diarrhoea, and hypoalbuminemia.

In 2008, Miyanishi et al. [60] investigated PAI gemcitabine (600, 800, 1000 mg/m^2^ on days 1 and 15 in three different cohorts) combined with the c.i. of 5-FU (300 mg/m^2^ on days 1–5 and 15–19 every 2 weeks) in 12 metastatic PC patients in a phase I clinical trial. All of the patients underwent super selective arterial embolization to alter the distribution of the blood flow into the pancreatic area, particularly through the larger pancreatic and the caudal pancreatic arteries. The ORR was 33.3%, while the mOS was 22.7 months. No severe toxicity was encountered.

In 2008, Sasada et al. [61] evaluated the PAI 5-FU (c.i. of 250 mg/m^2^/day for 7 days) and bolus infusion of cisplatin (5 mg/m^2^/day for 5 days) in 16 advanced PC patients in a phase II clinical study. The catheter was placed to allow the perfusion of both the pancreatic tumor and the liver. For the 12 patients with Stage Iva disease, the ORR was 58.3%, and the mOS was 22 months, while for the 4 patients with Stage IVb PC, the ORR was 0%. Hematologic and hepatic AEs were the most common toxicities, causing two patients to discontinue treatment.

In 2012, Tanaka et al. [62] enrolled 20 patients (2 patients with UICC stage III and 18 with IV) in a phase I/II study to analyze the efficacy and safety of PAI gemcitabine (1000 mg/m^2^) and 5-FU (increasing dose from 750 to 1000 mg/m^2^). Arterial perfusion was performed via the celiac artery after embolization of the pancreatic arteries originating from the SMA. The ORR, mOS, and PFS were 68.8%, 9.8 months, and 6 months, respectively. No technique-related complications and no severe AEs were reported, even when the dose of 5-FU was increased. The grade 3 side effects were neutropenia (15.8%) and thrombocytopenia (5.3%).

Published in 2012, a meta-analysis of six randomized controlled trials [63] evaluated the efficacy and safety of different PAI chemotherapy regimens compared to systemic treatments for advanced PC. This paper highlighted that PAI chemotherapy is more effective and has a lower risk of AEs than systemic chemotherapy. In detail, the ORR was higher in the PAI group (RR = 1.99, 95% CI: 1.50, 2.65; 58.06% versus 29.37%), as was the mOS (5–21 months versus 2.7–14 months). With regard to toxicity, AEs occurred in a lower percentage in the PAI group (RR = 0.72, 95% CI: 0.60, 0.87; 49.03% versus 71.33% in the systemic treatment group).

In 2014, Chen et al. [64] evaluated the safety and efficacy of PAI in a phase II study via an angiographic catheter placed into the arteries providing blood to the tumor with gemcitabine (1000 mg/m^2^) and oxaliplatin (100 mg/m^2^) every 4 weeks in 32 locally advanced PC patients. The ORR was 25%, the DCR was 65.6%, and the mOS was 10 months. No PAI-related side effects were observed; grade 3–4 gastrointestinal AEs occurred in 21.9% of patients.

In 2016, Liu et al. [65] retrospectively tested gemcitabine-based PAI chemotherapy (gemcitabine 1000 mg/m^2^ and oxaliplatin 100 mg/m^2^) in 354 patients (187 patients with UICC stage III and 87 with IV disease) using an angiographic catheter via the celiac artery and SMA. The mOS was 7 months, and no data about toxicity were reported.

In 2019, Qiu et al. [66] showed the clinical results of a retrospective evaluation of PAI chemotherapy in 115 patients (12 with stage II disease, 31 with stage III disease, and 72 with stage IV disease). The ORR was 5.2%, and the DCR was 62.6%, while the mOS was 4.9 months. These endpoints were significantly higher in patients with an ECOG score ≤ 1 and in those patients who received >1 sessions of PAI. Cerebral infarction, a sever AE, was reported after the technical procedure (0.9%). No other severe complications were reported.

#### 3.1.2. PAI as First Line Treatment with Systemic Chemotherapy

In 2006, Ikeda et al. [67] evaluated clinical results of combining PAI 5-FU (250 mg/day on days 1–5 every week in c.i.) with systemic gemcitabine (every week for 3 consecutive times) in 17 metastatic PC patients. An angiographic catheter was inserted into the celiac axis for allowing the chemotherapeutic perfusion of both the pancreatic tumor and the whole liver. The pancreatic arteries were super selectively embolized, excluding only the pancreatica magna and caudal pancreatic arteries flowing into the pancreatic parenchyma. The gastric and peripancreatic arteries were embolized by employing microcoils to prevent gastroduodenal toxicity due to the anticancer drugs. The ORR was 35% and 55% for the primary tumor site and liver metastases, respectively. The mean OS was 8.8 ± 1.5 months. No angiographic procedure-related AEs were documented. The most frequent side effects were hematologic alterations. Grade 3–4 non-hematologic AEs were observed in 23.5% of the patients, such as cholangitis, mild cerebral infarction, duodenal ulcer, and partial splenic embolization.

In 2013, Heinrich et al. [68] conducted a phase II trial testing a PAI combination (mitomycin C 8.5 mg/m^2^ and gemcitabine 500 mg/m^2^ on days 1 and 22) by inserting an angiographic catheter into the celiac artery with the systemic administration of gemcitabine monotherapy (500 mg/m^2^ on days 8 and 15) in 17 advanced PC patients. The ORR and mOS were 25% and 9.1 months, respectively. No procedure-related complications were reported. Hematological AEs were the most frequent ones, with 18 episodes of grade 3–4.

In 2013, Uwagawa et al. [69] reported the clinical outcomes of 35 PC patients (10 patients with UICC stage III and 20 with IV) who underwent PAI with Nafamostat Mesilate (4.8 mg/kg in c.i.) and systemic gemcitabine (1000 mg/m^2^ intravenously) on Days 1, 8 and 15 every 4 weeks in a phase II study. The mOS was 10.0 months, the ORR was 17.1%, and DCR was 88.6%. No technique-related complications were reported. Grade 3–4 hematological AEs were observed in 17% of patients.

#### 3.1.3. PAI as Second Line Treatment

In 2006, Barletta et al. [70] analyzed a PAI FLEC regimen as second-line treatment in 32 patients (7 with UICC stage III and 25 with IV) in a phase II clinical trial. The ORR was 21.9%, DCR was 58.8%, and mOS was 11.8 months from diagnosis. Treatment was not discontinued due to toxicity in any case.

### 3.2. Ongoing Clinical Trials

Recently, only a few clinical trials have been designed to better define the role of PAI chemotherapy in the treatment of advanced PC.

NCT02635971 is one of the most interesting ongoing clinical trials. It is an open label, randomized, double-arm, prospective phase II study to check the efficacy and safety of PAI versus intravenous infusion with gemcitabine plus oxaliplatin every 2 weeks, with 168 unresectable PC patients currently being enrolled.

Furthermore, NCT01665625 is an open label, randomized, double-arm, prospective clinical trial designed to check the safety and efficacy of PAI chemotherapy using an implanted percutaneous left subclavian artery port-catheter drug delivery system compared to systemic treatment in an estimated 90 estimated who are affected by advanced PC.

Finally, NCT03257033 is an open label, randomized, double-arm, prospective phase III study. All of the enrolled patients will receive intravenous nab-paclitaxel (125 mg/m^2^ over 30 min) and gemcitabine (1000 mg/m^2^ over 30 min) on days 1, 8, and 15 over he course of 4 weeks and radiation therapy for 4 months. Subsequently, patients will be randomized to receive PAI gemcitabine (1000 mg/m^2^ every week up to 8 administrations) using an intra-arterial catheter or to continue systemic chemotherapy for up to 16 weeks or until progression. Then, all of the subjects will receive systemic gemcitabine plus nab-paclitaxel or capecitabine.

## 4. General Conclusions and Future Perspectives

PC patients have a very poor prognosis due to the intrinsic chemoresistance of this type of tumor depending on: (a) the presence of a very dense, poorly vascularized, fibrotic envelope that involves pancreatic tumor area [13], (b) the poor vascularization of the pancreas, or (c) the high expression of the membrane-bound P-170 glycoprotein [18]. On the one hand, these factors hamper the systemic chemotherapeutic agents from reaching tumor cells in a sufficient enough amount to be effective and, on the other hand, the ATP-dependent drug efflux enzyme system quickly eliminates chemotherapeutic drugs from tumor cells, so standard therapeutic pharmacological options have limited success in PC [18].

Nowadays, a few therapeutic options are available for the treatment of PC that result in low RRs, short mPFS and mOS, and a high rate of severe side effects. The most effective treatment that is currently available for advanced PC is the FOLFIRINOX regimen [5]. This chemotherapeutic schedule has been proven to be more effective with respect to gemcitabine, though it led to an ORR of only 31.6% and an improvement in life expectancy of only 5 months with respect to gemcitabine [5]. In the 35% of PC patients who are affected by unresectable locally advanced disease at diagnosis [71], several clinical studies have demonstrated that FOLFIRINOX allows a resecability rate of about 26% to be obtained, and only 78% of patients receive an R0 resection [71,72]. It should be emphasized that all of these poor results have been obtained at the expense of a high rate of severe AEs, such as asthenia, vomiting, diarrhoea, peripheral neuropathy, and pancytopenia [5,71,73].

With regard to PAI chemotherapy, pharmacokinetic data have been evaluated in small series studies on animals and human beings, demonstrating the dose-dependent sensitivity of pancreatic tumor cells to this loco-regional treatment. In detail, the direct infusion of different chemotherapeutic agents such as gemcitabine and 5-fluorouracil to the tumor site allows for these drugs to achieve higher local concentrations while sparing healthy tissues [44,48,51].

On these bases, several clinical trials have tried to investigate PAI chemotherapy for unresectable locally advanced and metastatic PC with the aim of improving efficacy while limiting systemic toxicity.

To the best of our knowledge, most of the clinical studies reported in the scientific literature are phase II, with a single phase III study being available. The enrolled patient populations are affected by unresectable locally advanced and metastatic PC for liver involvement, and these subgroups are not distinguished in terms of clinical outcomes. Therefore, it is impossible to assess the effectiveness of the chosen experimental treatment for each clinical stage. Almost of all of the reported studies are characterized by a small patient population; for example, the phase III study only enrolled 138 patients, resulting in a subsequently low statistical weight. Furthermore, the administered chemotherapeutic agents are obsolete with respect to more recent treatments for PC. None of the reported trials evaluated either the current more effective treatments through PAI or a comparison with standard systemic therapy, except for the one phase III study. Finally, although it has been clarified that PC is a systemic disease since its inception [72], very few studies have been conducted to determine the combination of systemic treatment and PAI chemotherapy. The same studies did not compare this therapeutic strategy with standard chemotherapeutic treatment. Moreover, the enrolled population in these studies is not homogeneous because, as mentioned above, it included patients both with stage III and IV disease.

However, despite these important limitations, the reported data show promising results in terms of safety, reporting a very low rate of severe AEs experienced by the patients. Furthermore, PAI chemotherapy seems to provide important ORRs (see Table 1). As far as life expectancy, it is not possible to express an assessment, as the only phase III study compared the mOS between the two types of treatment, with favorable results.

Therefore, the role of PAI chemotherapy has not been established yet due to the lack of prospective, randomized, controlled, multicentre phase III clinical trials that compare this regional chemotherapy in combination with systemic one to systemic standard chemotherapy or systemic chemotherapy plus radiation therapy. In particular, patients with unresectable locally advanced PC may be more likely to benefit from this treatment with the aim of increasing their resecability rate.

Moreover, this review sheds light on the need to establish proper interventional oncological techniques and methodologies to define the best technical approach to treat the entire pancreatic tumor area.

Ongoing clinical trials might clarify these therapeutic issues. To this regard, our Interventional and Medical Oncology Unit is developing a phase II clinical trial consisting of PAI using modified FOLFIRINOX for patients who are affected by unresectable locally advanced PC with the aim of providing patients the most effective therapeutic regimen while also limiting severe systemic toxicity after its intravenous administration.

## Figures and Tables

**Table 1:** Compilation of references included in the review of the literature that evaluated PAI chemotherapy in advanced PC patients.

References	Type of Study	PAI Chemotherapy	Systemic Chemotherapy	DCR/RR * (%)	mPFS (mo.)	mOS (mo.)
Cantore et al. [54]	Phase II	FLEC	No	59/15 *	n.e.	9.9
Homma et al. [55]	Phase II	5-FU, cisplatin	No	73.9 *	n.e.	18.26 ± 10 **
Cantore et al. [56]	Phase III	FLEC (experimental group)	Gemcitabine (control group)	50 vs. 46 ^np^/14 * vs. 5.9 ^np^	n.e.	7.9 vs. 5.8 ^p^
Aigner et al. [57]	Phase II	Mitomycin, mitoxantrone, cisplatin	No	n.e.	n.e.	9
Mambrini et al. [58]	Phase II	FLEC	No	58.3/7.6 *	n.e.	9.2
Ishikawa et al. [59]	Phase II	Gemcitabine, 5-FU, cisplatin	No	50 *	n.e.	12
Tanaka et al. [42]	Pilot	5-FU, radiotherapy	No	70 *	n.e.	11
Miyanishi et al. [60]	Phase I	Gemcitabine, 5-FU	No	33.3 *	n.e.	22.7
Sasada et al. [61]	Phase II	5-FU, cisplatin	No	58.3 *	n.e.	22
Tanaka et al. [62]	Phase I/II	Gemcitabine, 5-FU	No	68.8 *	6	9.8
Liu et al. [63]	Meta-analysis	Different regimens	Yes (control groups)	58.06 vs. 29.37 ^p^	n.e.	5–21 vs. 2.7–14 ^p^
Chen et al. [64]	Phase II	Gemcitabine, oxaliplatin	No	65.6	n.e.	10
Liu et al. [65]	Retrospective	Gemcitabine, oxaliplatin	No	n.e.	n.e.	7
Qiu et al. [66]	Retrospective	No data	No	62.6	n.e.	4.9
Ikeda et al. [67]	Phase II	5-FU	Gemcitabine	45 *	n.e.	8.8 ± 1.5 **
Heinrich et al. [68]	Phase II	Mitomycin, gemcitabine	Gemcitabine	25 *	n.e.	9.1
Uwagawa et al. [69]	Phase II	Nafamostat mesilate	Gemcitabine	88.6/17 *	n.e.	10
Barletta et al. [70]	Phase II	FLEC	No	58.8/21.9 *	n.e.	11.8

* corresponds to RR (Response Rate); ** corresponds to median Overall Survival; np: not statistically significant difference; p: statistically significant difference.
